# Indigenous Children’s Knowledge About Non-timber Forest Products in Suriname

**DOI:** 10.1007/s12231-017-9400-4

**Published:** 2017-12-26

**Authors:** Tim van den Boog, Tinde van Andel, Janette Bulkan

**Affiliations:** 1 Forest Resources Management, Faculty of Forestry, University of British Columbia, Vancouver, BC Canada; 2 Naturalis Biodiversity Center, Leiden, Netherlands

**Keywords:** Traditional ethnobotanical knowledge, non-timber forest products, knowledge transmission, indigenous peoples, Suriname

## Abstract

Childhood and adolescence are important life stages for the acquisition of knowledge about non-timber forest products (NTFPs). We show at which stage in life traditional plant knowledge is learned and analyze whether cross-cultural ethnobotanical knowledge transmission takes place. We evaluate whether the degree of forest dependency influences ethnobotanical knowledge by comparing two indigenous communities in Suriname. Traditional knowledge was documented and vouchers collected during forest walks with adult informants. Questionnaires were completed by 74 schoolchildren (age 4 to 14) to capture their knowledge of names and uses of nine important NTFPs. We tested for knowledge differences by ethnicity and NTFP categories. Local names for NTFPs were analyzed to determine cross-cultural transmission of ethnobotanical knowledge. Children from the forest-dependent Trio community (*n* = 23) possessed similar knowledge of NTFPs as their more urbanized peers from Apoera (*n* = 51). NTFP uses were acquired at an earlier age than plant names. Food and commercial NTFP uses were better known than medicinal plant uses. Cross-cultural transfer of knowledge occurred between the two communities. NTFP knowledge of children appeared to be influenced more by the time they spent within the forest, either walking to school or walking to agricultural plots, than by the level of forest dependency or acculturation.

## Abstract (fr)

De jeugd en pubertijd zijn belangrijke levensfasen voor het verwerven van kennis over Niet-Hout Bosproducten (NTFP’s) bij bewoners van het Amazonegebied. Dit onderzoek had als doel te achterhalen in welke levensjaren traditionele plantenkennis wordt toegeëigend en of er ethnobotanische kennis wordt uitgewisseld tussen twee Inheemse gemeenschappen in Suriname. Verwacht werd dat de afhankelijkheid van het bos de hoeveelheid ethnobotanische kennis beïnvloedt. Tijdens boswandelingen met volwassen informanten is traditionele kennis over NTFPs gedocumenteerd en zijn er botanische specimens verzameld. Door 74 schoolgaande kinderen (4 tot 14 jaar) zijn vragenlijsten ingevuld om hun kennis te achterhalen over lokale namen en toepassingen van negen belangrijke NTFP soorten. Het verschil in kennis werd vergeleken tussen twee ethnische groepen en NTFP-categorieën (eetbaar en medicinaal). Lokale namen voor NTFP’s werden geanalyseerd om overdracht van ethnobotanische kennis tussen de gemeenschappen te bepalen. Tegen de verwachting in hadden kinderen uit de bosafhankelijke Trio-gemeenschap (*n* = 23) dezelfde kennis over NTFP’s als de meer verstedelijkte kinderen uit Apoera (*n* = 51). NTFP-toepassingen waren op jongere leeftijd beter bekend dan plantennamen. Eetbare en commerciële bosproducten waren beter bekend dan medicinale planten. Overdracht van kennis vond plaats tussen de twee gemeenschappen. De NTFP-kennis van kinderen bleek meer beïnvloed te worden door de tijd die ze in het bos besteden, dan door het niveau van bosafhankelijkheid of acculturatie.

## Introduction

The body of traditional knowledge about non-timber forest products (NTFPs) held by indigenous peoples has been declining over the past century. The influence of colonizing farmers, missionaries, conflicts overland rights, acculturation, and urbanization continue to cause social, economic, and environmental changes to indigenous communities and their territories (Kambel 2006; van Dam 2011; Voeks and Leony 2004; Zent and López-Zent 2004). Some losses of traditional ethnobotanical knowledge are triggered by restricted access to forests imposed by industries or governments that exclude local communities from the harvesting of NTFPs on their customary territories (Blaser et al. 2011). Other losses are due to changes in the livelihoods and lifestyles of indigenous peoples in which certain NTFPs are substituted by modern goods that are less time-consuming to prepare, more effective, and considered up-to-date (McCarter and Gavin 2015; Quave and Saitta 2016; Voeks and Leony 2004). Moreover, urbanization (e.g., the introduction of state schools, shops, and medical clinics) implies less time to be spent on traditional activities that involve NTFPs (Furusawa 2009; Reyes-García et al. 2009; Ruiz-Mallén et al. 2013; Zent 2009). On the other hand, influences from the outside have also proven to enhance certain traditional ecological knowledge (Quave and Saitta 2016). Overall, however, formerly important ethnobotanical knowledge tends to become superfluous and may be quickly forgotten or deliberately rejected when no longer used in (traditional) activities, thereby reshaping the forest dependency of local communities.

Byron and Arnold (1999) distinguish three levels that describe the degree of forest dependency of local communities: (1) the main source of a community’s livelihood depends on the surrounding forest. This category includes hunter-gatherers and subsistence farmers; (2) a community relies on nearby forests and its NTFPs for both subsistence and economic purposes through market sales; and (3) people are not dependent on the forest for their livelihood, but their economic welfare depends (partially) on the benefits derived from NTFPs. For communities that evolve through these categories, the importance of NTFPs changes from subsistence-oriented (use value) to economic importance (exchange value), hence changing the nature of certain ethnobotanical knowledge as it is passed on to younger generations.

Traditional knowledge is transmitted between generations in three ways (Cavalli-Sforza et al. 1986; Eyssartier et al. 2008; Ruiz-Mallén et al. 2013): (1) *vertical transmission*: between generations but within the genealogy of a family, (2) *horizontal transmission*: between peers of the same generation, and (3) *oblique transmission*: between generations but without familial ties. Whether acculturation leads to discontinued or disturbed knowledge transmission is still not conclusive. But cross-cultural knowledge transmission can also strengthen knowledge acquisition between people from different ethnic backgrounds (Furusawa 2009; Zent and López-Zent 2004).

Most knowledge about NTFPs is acquired during childhood and adolescence through playing, experiential participation, and observation, and during informal, traditional activities such as walks through the forest, storytelling, agricultural practices, rituals, and medicinal plant use (Hunn 2002; Ruiz-Mallén et al. 2013; Zent 2009; Zent and López-Zent 2004). Parents, peers, and older siblings play an important role in the transmission of ethnobotanical knowledge (Zarger 2002). Three to five year olds are already able to distinguish and identify various edible and non-edible plants (Quinlan et al. 2016), whereas by the age of 12, children can identify most of the important cultivated plants (Reyes-García et al. 2009; Zarger 2002; Zarger and Stepp 2004). For NTFPs, Zent (2009) shows that knowledge about NTFPs strongly increases until the age of 20. Disruption of knowledge transmission during childhood and adolescence can therefore reinforce the overall loss of ethnobotanical knowledge within indigenous communities.

Amerindian plant use in Suriname is well-documented (Ahlbrink 1931; Heemskerk et al. 2006; Heemskerk et al. 2007; Hoffman 2009; Ostendorf 1962; Plotkin 1986; Stahel 1944; van Andel and Ruysschaert 2011; van Andel et al. 2015). Still, for several indigenous areas of Suriname, traditional knowledge of NTFPs has never been documented. No data exist on the current transfer of traditional knowledge among Amerindians in the country’s rapidly changing society. This study compares children’s knowledge about NTFPs between an acculturated, predominantly Arawak village and a more forest-dependent Trio settlement in West Suriname. As only a 40-minute walk separates the two communities and their children attend the same school, this offers opportunities for cross-cultural exchange of ethnobotanical knowledge. We hypothesize that:

1. Trio children possess more knowledge about NTFPs than their peers from Apoera. We expect this because the Trio are more forest-dependent and have been less exposed to acculturation and urbanization, so they should have experienced less loss in traditional knowledge.
2. Cross-cultural knowledge transmission takes place between these two communities.
3. Nourishing and economically important NTFPs are better known than medicinal NTFPs by children from both communities.

## Materials and Methods

### Study Site and the Communities

**Fig. 1:**
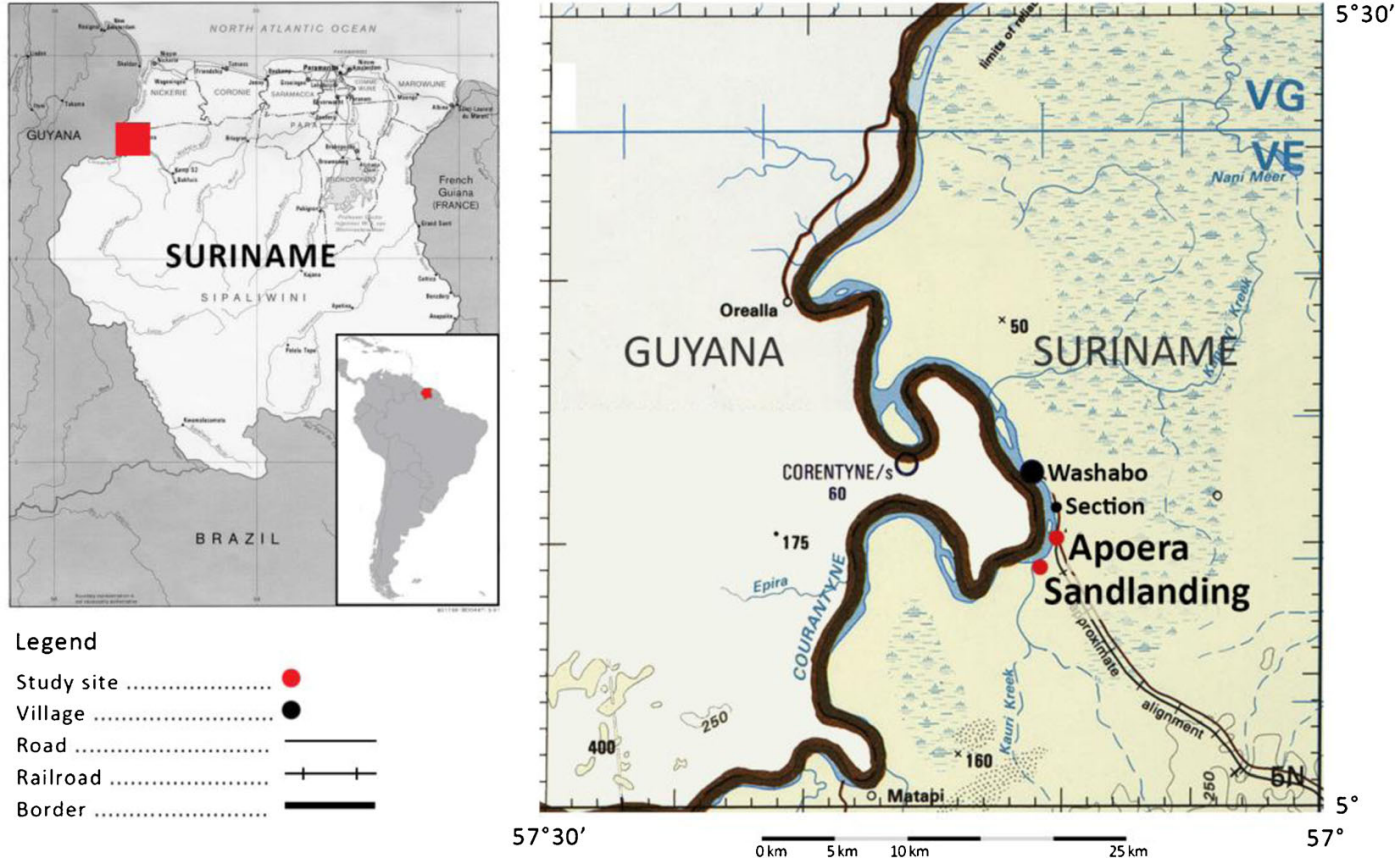
Study sites Apoera and Sandlanding along the Courantyne River, which borders Guyana and Suriname. Map modified from http://www.lib.utexas.edu/maps/tpc/txu-pclmaps-oclc-22834566_l-28a.jpg.

The Kabalebo jurisdiction of district Sipaliwini is located in the western part of Suriname and is home to the three indigenous villages—Apoera, Section, and Washabo—situated along the Courantyne River (Fig. 1). This research took place in Apoera (5° 11.43′ N and 57° 10.38′ W), a predominantly Arawak village, and Sandlanding (5° 9.81′ N and 57° 10.20′ W), a young Trio settlement that falls under the jurisdiction of Apoera. Access to larger villages entails a 7–10-hour drive east towards the capital Paramaribo, or a 120-kilometer (km) boat ride north to Nickerie. The study area, located in a tropical rainforest climate, has a mean annual temperature of 27 °C and an annual precipitation of 1895 mm. The ecosystem along the Courantyne River is characterized by tropical lowland forest vegetation, surrounded by highland forests and swamp forests.

The Arawak are the founders of the indigenous settlements along the Courantyne River. As a result of extractive industrial development in the 1930s (timber), 1940s (balata derived from the latex of *Manilkara bidentate* (A. DC.) A. Chev.), and 1970s (timber and bauxite), people from coastal Suriname and Guyana moved to West Suriname in search of employment (Romalo et al. 2009; Zschuschen 2011). As the non-locals were not allowed to settle in Washabo, they settled in Apoera, exposing the original community to other cultures and traditions. During the 1970s, several shops, a primary school, and other facilities were established. Although most resource-extractive activities stagnated during the Civil War in the 1980s, some newcomers settled in Apoera. Nowadays, Apoera’s approximately 1150 inhabitants are mainly Arawak, with some Warao and Carib Amerindians, speaking Guyanese Creole and Dutch Creole (Sranantongo) (VIDS 2008) (pers. comm. Capt. Lewis 2016). In 2005, 37% of the inhabitants were between 0 and 17 years of age (Romalo et al. 2009). Most inhabitants earn an income by working for logging companies (Greenheart and Nootje) or by selling NTFPs of which the tiger stingray (*Potamotrygon boesemani*) and crabwood oil (extracted from the seeds of *Carapa guianensis* Aubl.) are the most important (van den Boog pers. obs.). Traders from Paramaribo buy most of these NTFPs.

Between 2002 and 2005, about 10 Trio families settled in Sandlanding in order to obtain access to health care and education. The Trio families came from the remote island named Wanapan in the Courantyne River, roughly 200 km upstream from Apoera, where they depended entirely on their natural surroundings for their subsistence. Hence, most individuals within this community have been exposed to urban influences (acculturation, schooling, medical facilities, and shops) for 12 years. Currently, three of the 75 Trios in Sandlanding are employed at the sawmill of the Greenheart Company, 36 are primary schoolchildren, and the others make some money by selling seed jewelry, tiger stingrays, and other wildlife. Their subsistence and limited market sales are almost entirely based on traditional farming, hunting, and gathering of NTFPs. The Trio are thus more forest-dependent (second category of Byron and Arnold’s (2009) forest dependency categories) than their more acculturated close neighbors in Apoera (third category). All Trios speak the Trio language and some Sranantongo. Children are taught in the Dutch language at the primary state school in Apoera from age four.

The indigenous communities of Apoera, Washabo, and Section were assigned a collective piece of land (Communal Forest) by the government on which they can practice their traditional customs (e.g., housing, hunting, agriculture, logging, and gathering NTFPs). However, the Communal Forest covers only a small portion of the territory that is customarily and ancestrally used by these indigenous communities. Moreover, the area where the recently settled Trio live and hunt falls completely outside of the Communal Forest, but within the customary territory of Apoera, and is State owned under statutory law. Large areas that overlap customary territories have been allocated to international logging companies in this part of Suriname.

### Ethnobotanical Data Collection

This research was covered by UBC ethics certificate number H15-02527. Prior to the start of ethnobotanical fieldwork, a meeting was setup with the local authorities of Apoera and Sandlanding to request their consent according to Free, Prior and Informed Consent (FPIC) guidelines (University of British Columbia 2015). Fieldwork took place in March and April 2016 for 5 weeks. Forest walks were held with five women (three Trio and two Arawak) and eight men (two Trio and six Arawak) who were praised by village authorities and members for their ethnobotanical knowledge. Their personal data such as age, gender, and ethnicity were recorded (Phillips and Gentry 1993). During the walks, local plant names in both Trio and Arawak and their knowledge of NTFPs were documented. Photographs and voucher specimens were taken of the studied plants in accordance with the plant collecting permit issued by the Foundation for Forest Management and Production Control (SBB) in Suriname. The specimens were identified and deposited in the National Herbarium of Suriname (BBS) and Naturalis Biodiversity Center (L) at Leiden, the Netherlands. NTFPs without voucher material were identified by means of photographs, their local names, and recent literature on NTFP from the Guianas (van Andel 2000; van Andel and Ruysschaert 2011). Scientific names were verified through theplantlist.org.

### Questionnaires

In order to gather data on children’s NTFP knowledge, eight individuals from each class at the primary school were each asked to identify the names and uses of nine freshly collected NTFPs. Generally, four boys and four girls were randomly selected. All Trio children that were present were included since they formed a strong minority and would otherwise have been underrepresented. The local names of the nine NTFP species were provided in Trio and Arawak by the adult NTFP specialists during the forest walks. The selected species represented commonly known plants among the Trio and Arawak, were present in the immediate vicinity of both villages, and were easily recognizable in both fertile and sterile states. In addition to leaves, flowers and/or seeds (when present) and used plant parts were displayed as well. Age, ethnicity, and gender of all participants were recorded. One Trio adult translated for younger Trio children who had trouble understanding Dutch or Sranantongo. The NTFPs were divided into food, commercial, and medicinal plant categories. The details on the uses of NTFPs are not reported here but have been made fully available to the communities, as agreed in the FPIC contract with the communities. Local names were verified with data obtained during fieldwork and existing literature (Hoffman 2009; van ‘t Klooster et al. 2003; van Andel and Ruysschaert 2011). The language used to identify NTFPs was analyzed to record cross-cultural knowledge transmission between the communities.

### Data Analysis

To determine the level of knowledge of a participant, the children could score one point per correct answer for either the vernacular name or the use of an NTFP, so that a total of 18 points could be scored. Correctly identified NTFP characteristics (names and uses) were considered as a proxy for the NTFP knowledge of a participant. The totals were then transformed to percentages. Linear regression was used to model the relationship between NTFP knowledge and age. Variances were checked to be equal and independent sample *t* tests were then done to test for differences in scores between indigenous groups. Paired *t* tests were performed to analyze whether the means differed between dependent groups, such as the known uses and names of NTFPs and different NTFP categories (food/commercial and medicine). Statistical tests were carried out by using SPSS 24.0. Differences were considered significant when *p* < 0.05.

## Results

In total, 74 children (39 girls and 35 boys) participated in the questionnaire to identify names and uses of nine frequently occurring NTFP species known to both communities (Table 1). Ethnicity was given preference over gender as fewer Trio children attended the school, thereby shifting the 50:50 gender ratio. Of all participants, 51 were children from Apoera (predominantly Arawak with mixed Warao and Carib) and 23 were of Trio descent who inhabited Sandlanding. Six children decided not to participate in this study.

**Table 1:** LOCAL AND SCIENTIFIC NAMES OF THE NINE KEY NTFPS USED IN THIS RESEARCH.

Sranangtongo	Arawak	Trio	Surinamese Dutch	Scientific name	Family	Use
Kokriki	Barakaro	Weteu	–	*Ormosia costulata* (Miq.) Kleinhoonte	Fabaceae	Commercial
Krapa (siri)	Karaba	Karapa	Krapa	*Carapa guianensis* Aubl.	Meliaceae	Commercial/med.
Inginoto	Totoka	Tuhka	Brazielnoot^1^	*Bertholletia excelsa* Bonpl.	Lecythidaceae	Commercial/food
Rediloksi	Shimiri kuru	Kauru	Rode lokus^2^	*Hymenaea courbaril* L.	Fabaceae	Food
Slabriki	Yawahepesi	Pianaroy	Senna blad^1^	*Senna alata* (L.) Roxb.	Fabaceae	Medicinal
Sangrafu	Hokurishikaro	Oloke	Wenteltrap^2^	*Costus scaber* Ruiz & Pav.	Costaceae	Medicinal
Mokomoko	Yurika	Kurukuni	–	*Montrichardia arborescens* (L.) Schott	Araceae	Medicinal
Kwasibita	Kareudan	Malaria epi	Kwassiebitter^1^	*Quassia amara* L.	Simarubaceae	Medicinal
Busipapaja	Wanasoro	Ume	Man bospapaja^1^	*Cecropia sciadophylla* Mart.	Cecropiaceae	Medicinal

^1^van Andel and Ruysschaert (2011)

^2^van ‘t Klooster et al. (2003)

**Fig. 2:**
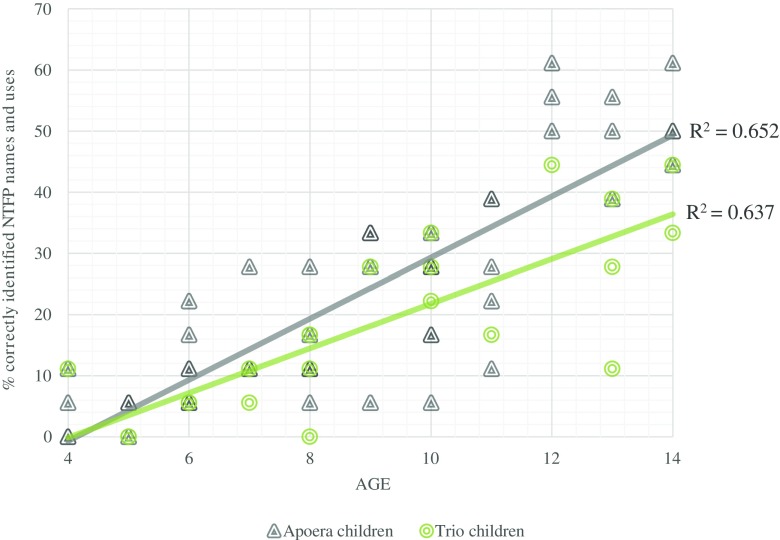
Linear regression of individual scores for children from Apoera (*n* = 51) and Trio (*n* = 23) children by age.

Linear regression analysis showed a strong relationship between age and correctly identified NTFP names and uses (*p* = 0.000 for both ethnic groups; Fig. 2). Although informants from Apoera scored slightly higher than Trio informants, there was no significant difference found in NTFP knowledge between children of both communities (*p* = 0.210).

**Fig. 3:**
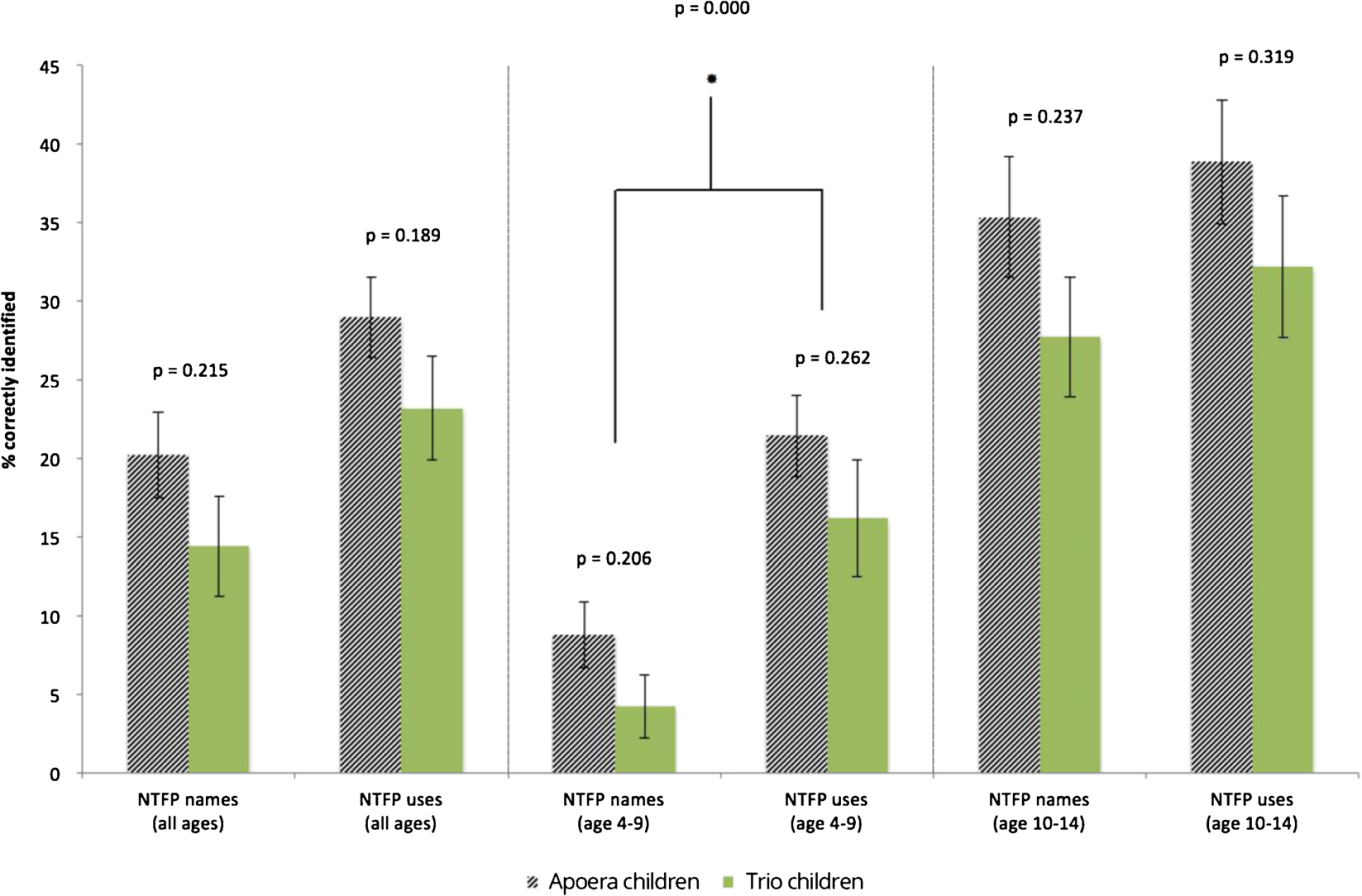
Comparison of correctly identified NTFP names and uses between children from Apoera and Trio children for all ages (*n* = 74), ages 4 to 9 (*n* = 42), and ages 10 to 14 (*n* = 32).

On average, Trio children were able to name 14% of the NTFPs, while children from Apoera named 20% correctly, but this difference was not significant (*p* = 0.215; Fig. 3). For NTFP uses, a similar pattern was seen: Trio children knew 23% of the NTFP uses, whereas children from Apoera knew 29%. The differences were not significant (*p* = 0.189; Fig. 3). When the groups were split up into two age classes (4 to 9 and 10 to 14), we did not find significant differences in NTFP knowledge between the children from both communities (Fig. 3). However, when the ethnic variances were disregarded, we found that younger children (aged 4 to 9) scored significantly higher at identifying NTFP uses (20%) than NTFP names (7%) (*p* = 0.000). For the older children, this difference was insignificant (*p* = 0.155).

**Fig. 4:**
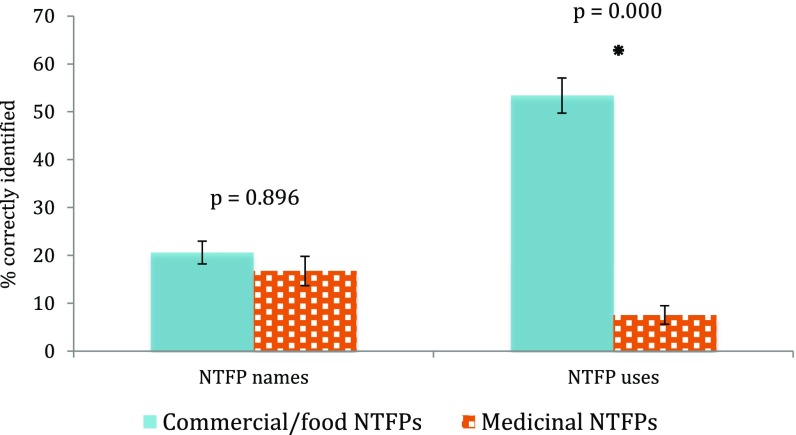
Comparison of identified NTFP characteristics (names and uses) for a group of commercial/food NTFPs and medicinal NTFPs for all participants.

When we further analyzed participants of both communities taken together, we found that correctly identified names for food/commercial and medicinal NTFPs did not significantly differ (*p* = 0.896; Fig. 4). However, uses of food/commercial species were significantly more often correctly identified (53%) than medicinal uses (8%) (see Fig. 4).

**Fig. 5:**
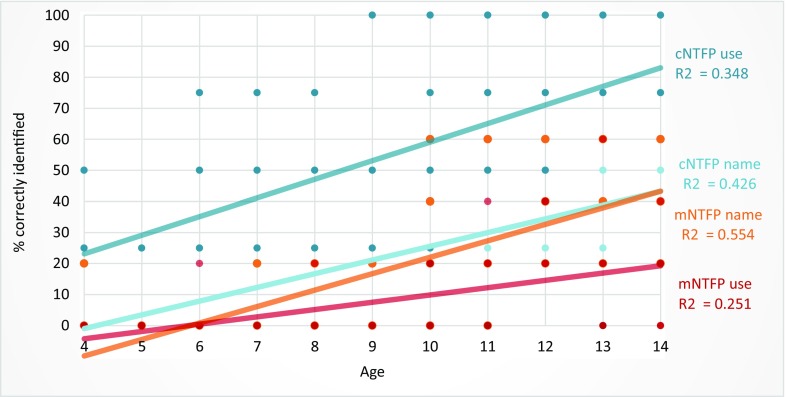
Linear regression of correctly identified medicinal NTFP (mNTFP) and commercial/food NTFP (cNTFP) names and uses by age. Each dot represents the participant’s percentage of a correctly identified NTFP characteristic (cNTFP use, cNTFP name, mNTFP use, mNTFP name).

When analyzed by age, we found that the uses of commercial/food NTFPs are understood by the youngest children, while knowledge of the names of commercial/food NTFPs and medicinal NTFPs appears to be acquired later. The knowledge acquired the latest were the uses of medicinal plants (Fig. 5). The youngest child from Apoera who named a medicinal NTFP correctly was 4 years old, while the youngest Trio was 9 years of age; both were boys. For correctly answering the use of a medicinal NTFP, the youngest child from Apoera was a 6-year-old boy, whereas from the Trio it was a 10-year-old girl.

Knowledge of commercial/food and medicinal NTFPs was compared between Apoerian and Sandlanding children. Overall, the children possessed similar NTFP knowledge except for the uses of medicinal plants, with which the Apoerian children were better acquainted (*p* = 0.041).

**Fig. 6:**
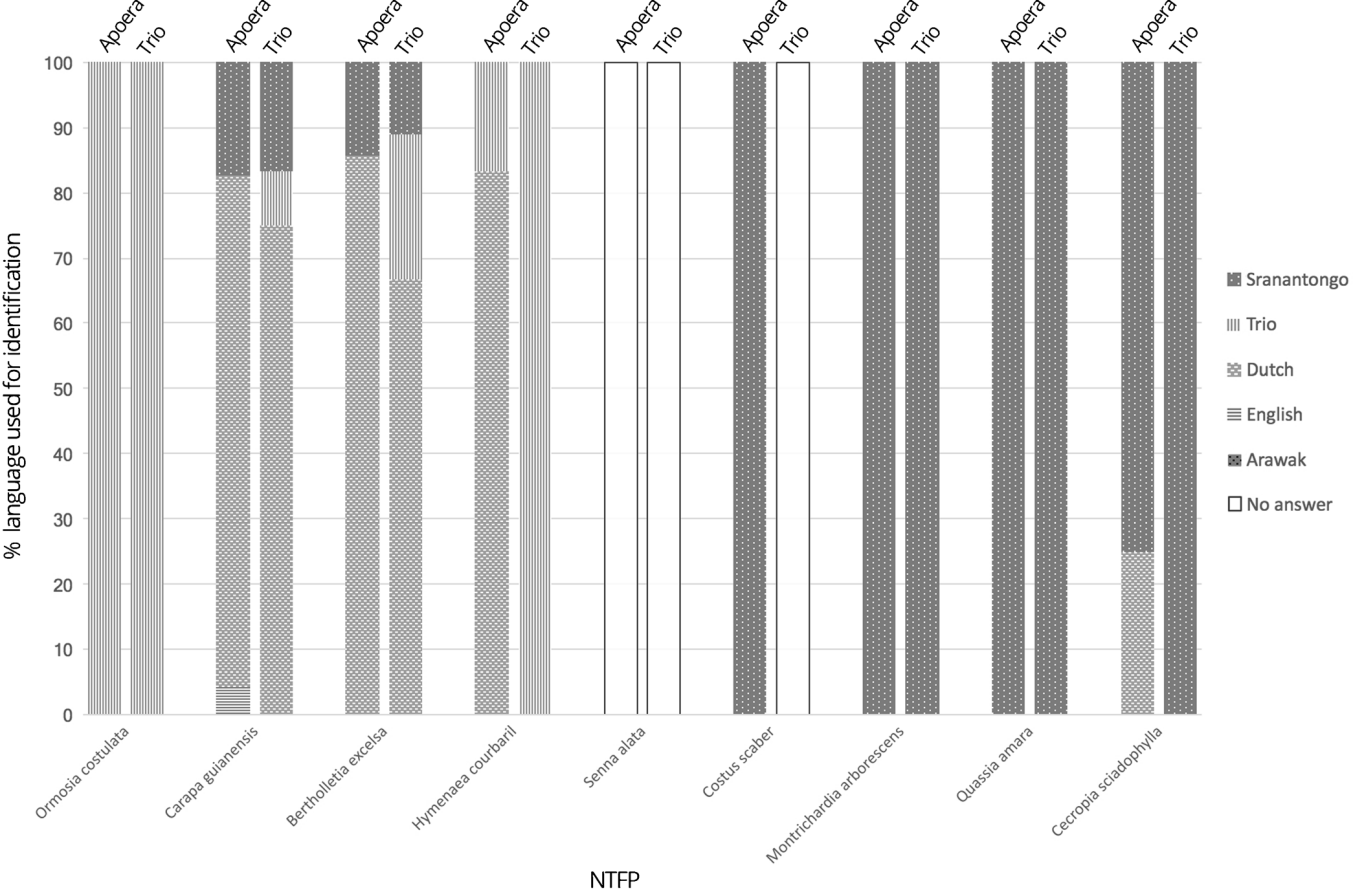
Different languages used by Apoera’s and Trio children for correctly identified NTFP names per species.

Figure 6 displays the languages used by the children when they correctly identified the species used in the questionnaire. Sranantongo was mostly used by both Apoera’s (50.5%) and Trio (46.2%) children, followed by Dutch (45.5% by children from Apoera, 38.5% Trio) and the Trio language (3.0% by Apoerians, 15.4% by Trios). English was used once to identify *Carapa guianensis*. Arawak was never used by any of the children. Although frequently occurring in and around the villages, *Senna alata* (L.) Roxb. was not correctly identified once—possibly because the specimen lacked the conspicuous yellow flowers. Neither was *Costus scaber* Ruiz & Pav. ever correctly identified by Trio children.

## Discussion and Conclusions

The main findings of this study are that the more acculturated children from Apoera possessed similar knowledge about NTFPs as the more forest-dependent Trio children. In both groups, edible and commercial NTFPs were better known than medicinal NTFPs. Cross-cultural knowledge transfer exists between Apoerians and Trios, but Trio children had acquired more knowledge from their Apoera peers than vice versa. As predicted, overall knowledge about NTFPs increased with age, as older children must have observed and experienced, both actively and passively, more traditional activities that involve NTFPs. We found that plant names and uses were acquired at different ages. Younger children were better acquainted with NTFP uses than with names, confirming that ethnobotanical knowledge transmission at a young age mostly happens through observation (Zent 2009). In the youngest group, NTFPs with commercial or nourishing value were better known than medicinal plants. Older children steadily increased their knowledge about NTFP uses and their corresponding local names, including the medicinal plants. Acquisition of plant knowledge seems thus more observer-oriented with a focus on food and commercial plants for younger children. Older children probably acquire more factual information on medicinal plants through active teaching. In contrast to food and commercially extracted NTFPs, medicinal plants are not used on a daily basis and their application requires specialized traditional knowledge (Zent and López-Zent 2004). Whereas food acquisition and consumption and trading happen constantly, medicinal plants are collected only when someone is ill. That means that even though basic medicinal plant knowledge is known to many individuals, children are likely to acquire this knowledge when they reach the age to join hunting and NTFP gathering trips or when relatives have been sick. Therefore, it would be interesting to follow-up on this research with adolescents.

### Disruption of Knowledge Transmission

As the Trio community’s subsistence was still entirely dependent on their natural environment until 12 to 14 years ago, we anticipated that their children would have more NTFP knowledge than their peers from Apoera. Apoera’s community has been exposed to acculturation and urbanization for several decades, factors that can erode vertical knowledge transmission (Reyes-García et al. 2010; Zent 2009). The Trio are still more reliant on traditional hunting, NTFP gathering, and agricultural practices than Apoera’s community, who are economically more prosperous and can therefore afford to buy food and tools in local shops. In contrast to our expectation, our comparisons of ethnobotanical knowledge showed that Trio children possess similar knowledge about NTFPs as Apoera children, or less, in the case of medicinal plant applications. Whether these results are influenced by the relatively small number of Trio participants or whether the loss of ethnobotanical knowledge is not so directly connected to acculturation and urbanization remains unknown*.* For instance, Quinlan and Quinlan (2007) confirmed that commercial occupations and modernized households were positively associated with medicinal plant knowledge. Ceuterick et al. (2011) also showed that urbanized migrants possess and apply traditional botanical remedies overseas. Rather than the level of education, gender, modernization, or any of the other usual suspects, the amount of plant knowledge can depend on one’s individual motivations, experiences, and personality (Mathez-Stiefel and Vandebroek 2012).

**Fig. 7:**
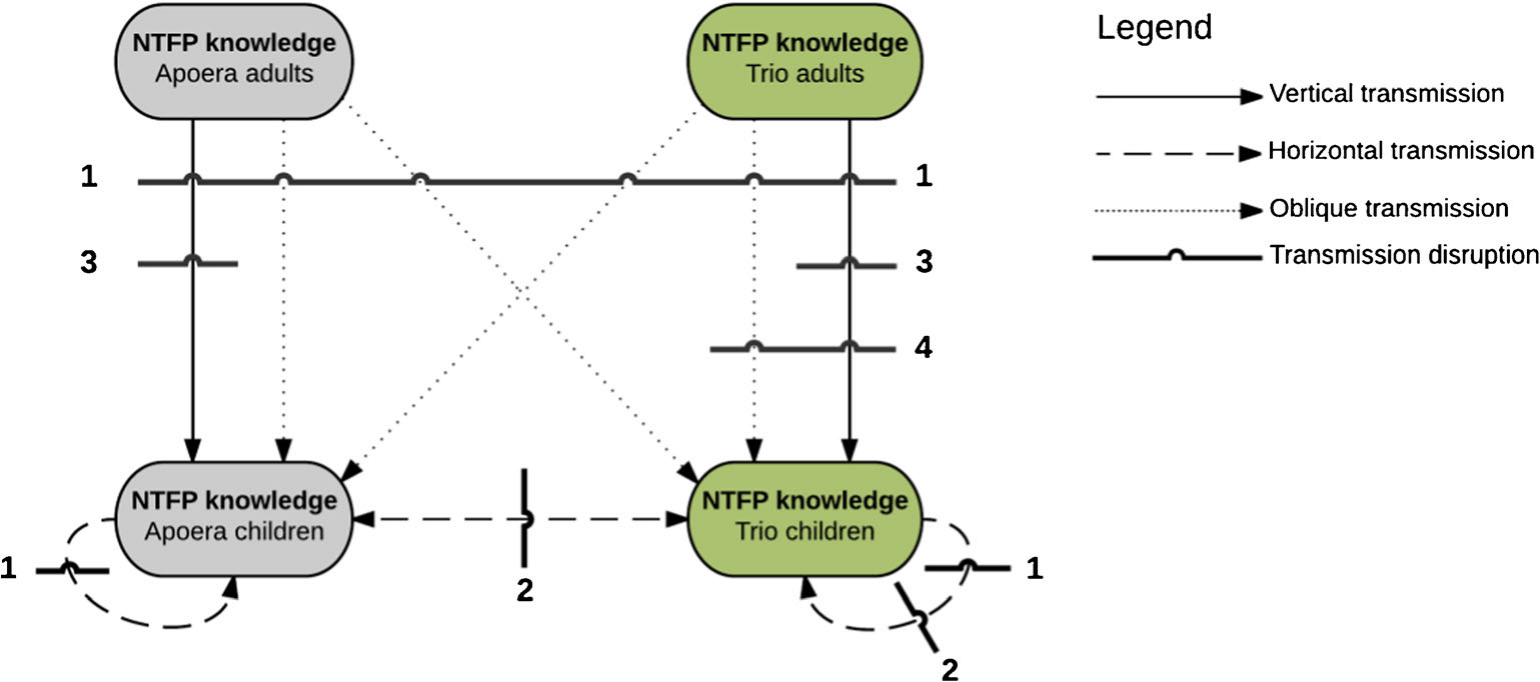
Model of transmission disruptions of NTFP knowledge from adults to children and between children from Apoera and the Trio community in Sandlanding. Line 1: School limits traditional teaching occasions. Line 2: School bus limits forest walks. Line 3: Distant and diminishing agricultural plots limit forest walks. Line 4: Migration from different environment limits local plant knowledge.

Based on our study results, we have created a model that visualizes NTFP knowledge transmission and disruption (Fig. 7). Several studies have shown that attending state schools reduces the body of traditional knowledge that children possess (Reyes-García et al. 2009; Ruiz-Mallén et al. 2013). Children spend less time with their peers, siblings, and adults in the community, when traditional knowledge could have been acquired during daily activities. State schools in Surinamedo not include the teaching of traditional practices in their curricula, which implies that schooling hours disrupt vertical, horizontal, and oblique traditional knowledge transmission (Fig. 7, line 1).

The journey to and from school is also an important moment for children to become acquainted with forest products through playing and interaction with peers (Ros-Tonen et al. 1998; van Andel 2000). In our case, Trio children are collected and returned to Sandlanding by a school bus, which eliminates their daily forest walk and thereby possibly disrupts horizontal traditional knowledge transmission (Fig. 7, line 2), so children from the two communities do not spend much time together after school. The school bus also limits the opportunity for cross-cultural horizontal knowledge transmission after school hours, as the Trio pupils are directly brought home after school. However, we observed that children still have the rest of the day to interact with their siblings, parents, and other community members, during which traditional knowledge is actively and passively passed on.

The Trios’ agricultural plots are located more than 10 km from their settlement. Practically, this means that adults (usually the women) charter an Apoerian car owner to take them to their fields. It is economically and spatially inconvenient to bring children along in a car that could also be filled up with other adults and agricultural produce (mainly cassava). Agricultural plots close to the village create more opportunities for children to acquire ethnobotanical knowledge on their way to their gardens (Reyes-García et al. 2010). In Apoera, it has become less common to have familial agricultural plots, leading to discontinued NTFP knowledge transmission. For both children groups, this results in disrupted vertical traditional knowledge transmission (line 3 in Fig. 7).

Another explanation for the comparable NTFP knowledge for both children groups is the fact that the Trios migrated from an ecologically different environment. The Trio adults explained that they had grown up in “a different flora, of which they learned all the traits and practices.” Trio women repeatedly mentioned that there was a wider variety of delicious fruits and herbs in their birthplace (Kwamalasumutu) in southern Suriname. It is likely that the Sandlanding Trio lack knowledge of plants in West Suriname. As a result, the vertical and oblique transmission of ethnobotanical knowledge to their children is limited to species that occur in both ecosystems and/or cultures (line 4, Fig. 7). Figure 7 shows that the Trio children experience more knowledge transmission disruptions than their Apoerian peers.

### Cross-Cultural Knowledge Exchange

The local names reported by children showed that NTFP knowledge transmission took place between the two communities. Children from Apoera did not mention any Arawak names. This was not surprising as only some elderly people still speak the Arawak language. Trio children identified three commercial and nourishing NTFPs usually in their own language (wetei, *Ormosia costulata*; kauru, *Hymenaea courbaril*; tuhka, *Bertholletia excelsa*), while the other names were usually given in Sranantongo or Surinamese Dutch. NTFP uses were mostly described in Dutch, likely because questions were asked in Dutch (or translated to Trio when necessary). Children from Apoera correctly identified the seeds of *Ormosia costulata* (the most important commercial vegetal NTFP for this Trio community) as “weto,” using the Trio name instead of the Sranantongo name “kokriki.” The fact that Trio children mostly knew Sranantongo or Dutch names and children from Apoera knew one Trio name indicates that cross-cultural NTFP knowledge transmission takes place between these communities, especially from the community of Apoera to the Trio. Further research could clarify whether this happens through direct horizontal knowledge transmission between the children, or through other ways. Research could then also be extended to a wider variety of NTFPs.

There is an opportunity for a traditional teaching program, since state schools at present do not include this in their curricula and yet children were eager to know more about the NTFPs presented in the questionnaire. To some, it will be of great use as many families still largely depend on natural resources for their subsistence. Because the curriculum is developed by the state, such a program would have to be approved and, if possible, funded by the government. The development and execution of a traditional teaching program, however, should be carried out by local people with sufficient knowledge of the locally relevant NTFP practices.
